# Determinants of stunting among children aged 0–59 months in Nepal: findings from Nepal Demographic and health Survey, 2006, 2011, and 2016

**DOI:** 10.1186/s40795-019-0300-0

**Published:** 2019-08-05

**Authors:** Ramesh P. Adhikari, Manisha Laxmi Shrestha, Ajay Acharya, Nawaraj Upadhaya

**Affiliations:** 1 Padma Kanya Multiple Campus, Tribhuvan University, Kathmandu Nepal and Helen Keller International Nepal, Lalitpur, Nepal; 2 FHI360, Kathmandu, Nepal; 3 University of Melbourne, Melbourne, Australia; 4 Transcultural Psychosocial Organization Nepal, Kathmandu, Nepal

**Keywords:** Children, Stunting, Nepal, Determinants

## Abstract

**Background:**

Stunting is one of the most commonly used indicators of child nutrition and health status. Despite significant efforts by the government and external development partners to improve maternal and child health and nutrition, stunting is consistently high in Nepal. This paper assesses the potential determinants of stunting among children aged 0–59 months using the last three successive Nepal Demographic and Health Surveys (NDHS).

**Methods:**

We used three nationally representative cross-sectional household surveys, known as the NDHS- 2006, 2011 and 2016. Logistic regression was used to identify the potential determinants of stunting. The sub sample for this study includes *n* = 5083 in 2006, *n* = 2485 in 2011, and *n* = 2421 in 2016.

**Results:**

Rates of stunting decreased from nearly 50% in 2006 to about 36% in 2016. The prevalence of stunting was higher among children from larger families (51.0% in 2006, 41.1% in 2011, 38.7% in 2016), poor wealth quintile households (61.2% in 2006, 56.0% in 2011, 49.2% in 2016), and severely food insecure households (49.0% in 2011, 46.5% in 2016). For child stunting, the common determinants in all three surveys included: being from the highest equity quintile (OR: 0.58 in 2006, 0.26 in 2011, 0.28 in 2016), being older (OR: 2.24 in 2006, 2.58 in 2011, 1.58 in 2016), being below average size at time of birth (OR: 1.64 in 2006, 1.55 in 2011, 1.60 in 2016), and being affected by anemia (OR: 1.32 in 2006, 1.59 in 2011, 1.40 in 2016).

**Conclusions:**

This study found that household wealth status, age of child, size of child at time of birth, and child anemia comprised the common determinants of stunting in all three surveys in Nepal. Study findings underscore the need for effective implementation of evidence-based nutrition interventions in health and non-health sectors to reduce the high rates of child stunting in Nepal.

**Electronic supplementary material:**

The online version of this article (10.1186/s40795-019-0300-0) contains supplementary material, which is available to authorized users.

## Background

Child malnutrition remains a major public health problem globally. Malnutrition can take the form of stunting (height-for-age and/or weight-for-height) and underweight (weight-for-age); however, stunting is the primary form of malnutrition seen throughout the world [1, 2]. Stunting in early life is associated with adverse functional consequences, including poor cognition and educational performance, low adult wages, lost productivity, and chronic disease when accompanied by excessive weight gain later in childhood [3–5]. While the consequences of stunting are clear, its causes are more complex [6]. Direct factors contributing to stunted growth and development include poor maternal health and nutrition, inadequate infant and young child feeding practices, micronutrient deficiencies, and infections [7]. The Sustainable Development Goals (SDG) identified stunting, along with other nutrition indicators, as a target to reduce global malnutrition; meeting this goal demands renewed and concerted efforts focused on disparity [8].

Globally, approximately 155 million children under 5 years suffer from stunting, with an estimated one million associated child deaths annually. Among the regions of the world, Africa and Asia share the highest burden of all forms of malnutrition. In 2017, nearly two out of every five stunted children lived in Southern Asia [9]. Nepal is one of only ten countries in the world where more than half of children under five suffer from some form of malnutrition [10]. According to the 2016 Nepal Demographic Health Survey, 36% of children under five suffered from stunting. Previous studies in Nepal have identified several common determinants of stunting, including child sex, age, birth weight, birth order, number of siblings, wealth index, mother’s education, mother’s body mass index, and access to health care [11–13]. The Nepal government identified nutrition as one of the key priority areas for national development. In 2012, the Nepal government developed a multi-sector nutrition plan involving different line ministries to implement an evidence-based nutrition intervention throughout health and non-health sectors. As a result, Nepal has made significant gains in health and nutrition indicators, including stunting, despite being in social, economic and political transition [14], and being one of the poorest countries in South Asia. Nevertheless, decreases in stunting stalled between 2012 and 2017. To meet the global target by 2025, Nepal needs to continue to reduce stunting by 3.9% annually [15].

The purpose of this paper is to determine the prevalence and associated factors of child stunting in the last 15 years in Nepal using nationally representative data from the Nepal Demographic and Health Surveys (NDHS) 1996–2016. While previous studies attempted to explore determinants of stunting, each only reported data from a single year. Currently, there is insufficient evidence on trends and determinants of stunting from nationally representative surveys in Nepal, thus limiting long-term policy formulation and planning. Hence, assessing the trends and factors associated with stunting will provide insights into the effectiveness of the implemented program and will assist policy makers and program developers in designing new and effective programs that target groups most at-risk.

## Methods

This paper uses data from the 2006, 2011, and 2016 NDHS, a nationally representative cross-sectional household survey. This dataset includes information on a variety of health topics, as well as socio-economic and demographic information. The risk factors have been classified into demographic factors, maternal nutrition factors, and child nutrition factors. The NDHS surveys used a two stage selection process; initially, enumeration areas (clusters) were selected with probability proportion-to-size (PPS) methodology and next, households were selected with an equal probability systematic selection from each sample cluster. The sampling details for this survey have been documented in the full NDHS report [16]. In 2006, height and weight information for a total of 5295 children was collected from 8707 interviewed households. Similarly, in 2011, 2603 children’s height and weight information was collected from 10,826 interviewed households, and in 2016, a total of 2428 children’s information was collected from 11,040 interviewed households. Some cases were excluded because their height and weight measurements were outliers (*n* = 212 in 2006, *n* = 118 in 2011, and *n* = 7 in 2016). Thus, the final sample sizes for the analyses in this paper were *n* = 5083 in 2006, *n* = 2485 in 2011, and *n* = 2421 in 2016. [16].

Stunting, the single outcome variable, was measured based on height-for-age z-scores <− 2 standard deviation. The NDHS calculated the height-for-age z-scores based on the 2006 World Health Organization (WHO) growth standards. These scores were used for the analysis [17]. Based on the local context and prior studies, household, maternal, and child characteristics were included as potential determinants of stunting in Nepal. Household characteristics included family size, headship of the household, caste/ethnic group (Dalit, Muslim, *Janajati*, other *Terai* caste, Brahmin/Chhetri, others), wealth quintile (based on NDHS wealth quintiles), place of residence (urban or rural), ecological zone (mountain, hill, or *terai*), household food security status (food secure, mildly food secure, moderately food insecure, or severely food insecure), and access to drinking water and a toilet (improved or unimproved). Maternal characteristics included the mother’s age, years of schooling, employment status (currently employed or not), BMI (underweight with a BMI < 18.5, or not), anemia (anemic or not), and number of living children. Likewise, child characteristics included age and sex of the child, birth order, size at time of birth (average or larger or below average), and anemia (anemic or not).

The NDHS used the same questions as the Household Food Insecurity Scale (HFIAS), which was originally developed by the United States Agency for International Development (USAID) Food and Nutrition Technical project to measure household food security status. These questions focused on the severity and frequency of food insecurity in the household units based on the 30 days preceding the survey. Based on responses to these questions, four categories were developed based on the HFIAS measuring guidelines: food secure, mildly food secure, moderately food insecure and severely food insecure [18]. Access to piped drinking water was defined as “improved” water access, while all other sources were defined as “unimproved”. Likewise, access to a toilet was defined as “improved” if the households had flush/pour toilets to piped sewer systems, septic tanks and/or pit latrines, ventilated improved pit latrines with slabs, and/or composting toilets. Access to other types of toilets were considered “unimproved”.

The NDHS collected blood samples to measure mother and child anemia using HemoCue. Children were considered anemic if their hemoglobin levels were < 11.0 g/deciliter. Likewise, mothers were considered anemic if their hemoglobin levels were < 12.0 g/deciliter for non-pregnant mothers and < 11.0 for pregnant mothers. Mothers were included if they had ever been married, were not currently pregnant, and had not given birth in the previous 2 months. Exclusion criteria included outliers in BMI, height, and weight measurements [16]. For the national representation of the sample, the analysis was performed by applying sampling weights. Descriptive, univariate, bivariate, and multivariate analyses was performed. Mean z-scores and standard deviations were calculated for height-for-age. Logistic regression models were used to identify the possible determinants of stunting. All analyses were performed in Stata version 14.

## Results

This study included 5083 children from the 2006 NDHS, 2485 children from the 2011 NDHS, and 2421 children from the 2016 NDHS. In all three surveys, more than a quarter of the children were from *Janajati* caste/ethnic groups, more than half were from the *Terai* region, and about 60% were between 24 and 59 months. With regard to mothers’ education, more than half of the mothers sampled had no schooling in 2006 and 2011; however, this was down to 36% in 2016 (Table 1).

**Table 1:** Socio-economic and demographic characteristics of children under 5 years and their mothers

Background characteristics	2006		2011		2016	
	%	N	%	N	%	N
Caste/ethnicity						
Dalit	15.5	788	17.7	440	14.1	342
Muslim	5.6	286	5.9	147	6.8	164
*Janajati*	31.6	1608	32.0	794	27.3	661
Other *Terai* caste	14.0	713	9.3	230	19.8	479
Brahmin/Chhetri	28.4	1444	29.5	734	26.0	629
Other	4.8	244	5.6	140	6.0	146
Wealth quintile						
Poorest	25.3	1285	25.8	640	20.5	496
Second poorest	21.4	1086	20.5	510	21.8	528
Middle	20.3	1032	23.4	582	22.7	549
Second richest	18.1	921	16.9	421	21.7	526
Richest	14.9	759	13.4	332	13.3	322
Ecological Zone						
Mountain	8.4	425	7.9	196	7.0	170
Hill	41.3	2100	39.9	991	36.2	876
*Terai*	50.3	2558	52.2	1298	56.8	1375
Year of schooling of mother						
No schooling	61.4	3119	50.5	1256	36.0	910
1–5 years schooling	16.9	858	17.8	442	19.0	449
6–9 years schooling	15.5	788	18.7	464	23.9	563
10 and above years of schooling	6.3	318	13.0	323	21.1	499
Age of child						
Less than 6 months	9.1	474	9.2	228	9.0	218
6–11 months	9.2	484	9.9	247	10.4	251
12–23 months	18.7	970	19.6	489	21.1	512
25–49 months	63.1	3155	61.3	1521	59.5	1440
Sex of child						
Boys	51.2	2604	51.2	1273	52.0	1258
Girls	48.6	2479	48.8	1212	58.0	1163
Total	100.0	5083	100.0	2485	100.0	2421

### Prevalence of stunting

The mean z-scores for stunting (height-for-age) showed slight improvement between 2006 and 2016 (− 1.92 in 2006 and − 1.29 in 2016). Likewise, the proportion of child stunting decreased from 49.3% in 2006 to 35.8% in 2016. The proportion of stunting was higher among larger families, children from the Dalit caste/ethnic group, households from the poorest wealth quintile, children residing in rural and mountain regions, and children from severely food insecure households (Table 2).

**Table 2:** Prevalence of stunting (<−2SD) among children aged 0–59 months

	2006		2011		2016	
	%	N	%	N	%	N
Total (mean z score and SD)	49.3 (−1.93; 1.3)	5083	40.5 (−1.66; 1.4)	2485	35.8 (−1.29; 4.9)	2421
*Household characteristics*						
Family size						
Less than 5	43.2	1247	38.9	693	29.3	727
5 and above	51.0	3836	41.1	1792	38.7	1694
Headship of the households						
Male	48.7	4052	41.2	1828	36.2	1652
Female	50.9	1031	38.7	657	35.0	769
Caste/ethnicity						
Dalit	56.5	788	47.0	440	38.7	342
Muslim	56.6	286	31.0	147	37.2	164
*Janajati*	44.9	1608	40.4	794	32.2	661
Other *Terai* caste	50.3	713	45.9	230	42.3	479
Brahmin/chhetri	47.8	1444	36.8	734	34.6	629
Other	48.5	244	41.0	140	28.3	146
Wealth quintile						
Poorest	61.2	1285	56.0	640	49.2	496
Second poorest	54.4	1086	45.7	510	38.7	528
Middle	50.6	1032	34.5	582	35.7	549
Second richest	39.7	921	30.5	421	32.4	526
Richest	30.5	759	25.8	332	16.5	322
Place of residence						
Urban	35.8	619	26.7	217	32.0	1280
Rural	51.0	4464	41.8	2268	40.2	1141
Ecological Zone						
Mountain	61.2	425	52.9	196	46.8	170
Hill	50.2	2100	42.1	991	32.3	876
*Terai*	46.2	2558	37.4	1298	36.7	1375
Household food security status						
Food secure	NA		33.2	1061	29.2	991
Mildly food insecure	NA		41.2	305	35.9	557
Moderately food insecure	NA		45.6	577	42.0	623
Severely food insecure	NA		49.0	542	46.5	250
Access of drinking water						
Unimproved	51.2	1296	48.9	361	43.3	112
Improved	48.4	3787	39.1	2124	35.5	2309
Access of toilet						
Unimproved	53.9	3659	45.0	1415	48.9	581
Improved	36.9	1424	34.6	1070	31.7	1840
*Maternal characteristics*						
Age of mother						
15–19	33.6	333	27.9	170	37.4	194
20–24	45.4	1761	39.8	908	32.5	863
25–29	49.2	1595	38.7	740	34.8	767
30 and above	58.6	1394	46.7	667	41.6	597
Years of schooling of mother						
No schooling	57.3	3119	46.9	1256	45.3	910
1–5 years schooling	45.7	858	41.7	442	36.4	449
6–9 years schooling	31.9	788	32.1	464	31.7	563
10 and above years of schooling	20.1	318	25.9	323	22.7	499
Number of living children						
Up to 1 children	34.0	1104	31.9	680	28.0	725
2 children	46.0	1614	38.5	751	32.0	841
3 and more children	58.3	2365	47.5	1054	46.2	855
Mother Employment						
No	42.7	1580	37.5	1116	31.7	1240
Yes	52.0	3503	42.9	1369	40.2	1181
Mother BMI						
less than 18.5/underweight	47.2	3797	38.6	1935	33.6	1901
18.5 and above	54.7	1284	47.1	469	44.5	452
Mother anemia						
No	48.1	3027	39.8	1476	35.4	1271
Yes	50.9	2023	41.2	902	35.8	1074
*Child characteristics*						
Age of child						
Less than 6 months	11.5	474	19.4	228	13.5	218
6–11 months	23.65	484	15.9	247	18.9	251
12–23 months	47.5	970	34.8	489	37.4	512
25–49 months	59.2	3155	49.5	1521	41.6	1440
Sex of child						
Boys	48.6	2604	41.4	1273	36.0	1258
Girls	49.7	2479	39.5	1212	35.7	1163
Birth order						
First	40.6	1378	34.2	781	31.4	791
Second	44.9	1266	39.2	592	30.1	657
Third and above	56.1	2439	45.6	1112	43.3	973
Size at the time of birth						
Average or larger	46.9	4109	38.4	2061	34.0	2031
Below average	59.1	974	50.7	424	45.5	391
Anemia						
No	53.6	2284	41.4	1172	34.7	1022
Yes	53.9	2191	45.2	1008	40.9	1137

Note: Only % for stunning are reported for each category. So, the total will not be 100% because for each category % of non-stunning are not included in this table, see more on Additional files 1, 2 and 3.

In all three surveys, the proportion of child stunting decreased as the number of years of mothers’ schooling increased. For example, the prevalence of child stunting was 45.3% among children whose mothers had no education, compared with 22.7% among children whose mothers had 10 or more years of schooling in 2016. Furthermore, the increases the number children living in a household also increase the likelihood of stunting. For instance, in 2016, the prevalence of stunting was 28% in single child households compared with 46.2% for the households with 3 or more children. The chances of stunting were also greater among children with anemic mothers. Child characteristics such as age, sex, birth order, birth size, and hemoglobin level were found to be determining factors for stunting in all three surveys. Stunting increased with increases in age and birth order. Likewise, small size at birth, female gender, and low level of blood hemoglobin (anemia) increased the likelihood of stunting in children (Table 2).

### Factors associated with stunting

The adjusted odd ratios in Table 3 show that in all three surveys, wealth quintile, age of child, size of child at birth, and anemia were significant determinants of stunting in children. In the adjusted model, we adjusted for household, maternal, and child characteristics including family size, headship of the household, caste/ethnicity, wealth quintile, place of residence, household food security status, access to drinking water and a toilet, mother’s age, mother’s years of education, number of living children, mother’s employment status, mother’s BMI and anemia status, age and sex of child, birth order, size of child at time of birth, and child anemia status. In all three surveys, children in the richer and richest wealth quintile households were less likely to be stunted compared with children from the poorest quintile households. Likewise, in all three surveys, older children were more likely to be stunted than younger children (OR: 2.24 in 2006, 2.58 in 2011, 1.58 in 2016). Children who were below average size at time of birth were 1.6 times more likely to be stunted in all three surveys compared with children who were average size or larger at time of birth (OR: 1.64 in 2006, 1.55 in 2011, 1.60 in 2016). Similarly, children who suffered from anemia were more likely to be stunted in all three surveys (OR: 1.32 in 2006, 1.59 in 2011, 1.40 in 2016) (Table 3).

**Table 3:** Determinants of stunting in children aged 0–59 months

Background characteristics	Odd ratios of stunting (height for age < −2SD)					
	2006		2011		2016	
*Household characteristics*	Unadjusted (OR, P/ CI)	Adjusted (OR, P/ CI)	Unadjusted (OR, P/CI)	Adjusted (OR, P/CI)	Unadjusted (OR, P/CI)	Adjusted (OR, P/CI)
Family size	1.37** [1.16–1.62]	1.15 [0.95–1.38]	1.01 [0.86–1.40]	0.87 [0.62–1.21]	1.52** [1.22–1.90]	1.39* [1.06–1.83]
Headship of the households						
Male (R)						
Female	1.09 [0.91–1.31]	1.01 [0.82–1.24]	0.90 [0.71–1.14]	0.85 [0.64–1.14]	0.95 [0.77–1.17]	1.06 [0.84–1.35]
Caste/ethnicity						
Dalit(R)						
Muslim	1.00 [0.74–1.35]	1.21 [0.82–1.80]	0.50 [0.30–0.85]	0.55 [0.27–1.14]	0.94 [0.60–1.47]	1.04 [0.61–1.78]
*Janajati*	0.63 [0.48–0.83]	0.73 [0.53–0.99]	0.76 [0.56–1.04]	0.89 [0.61–1.29]	0.75 [0.53–1.08]	1.01 [0.69–1.47]
Other *Terai* caste	0.78 [0.59–1.03]	0.96 [0.69–1.33]	0.95 [0.63–1.43]	1.56 [1.04–1.33]	1.16 [0.81–1.66]	1.18 [0.74–1.91]
Brahmin/chhetri	0.70 [0.57–0.89]	0.94 [0.69–1.26]	0.65 [0.51–0.85]	0.80 [0.58–1.09]	0.84 [0.60–1.17]	1.08 [0.72–1.61]
Other	0.72 [0.35–1.50]	0.80 [0.36–1.77]	0.78 [0.48–1.29]	0.96 [0.39–2.37]	0.63 [0.37–1.05]	0.60 [0.26–1.36]
Wealth quintile						
Poorest (R)						
Second poorest	0.76** [0.63–0.92]	0.87 [0.65–1.18]	0.66** [0.50–0.88]	0.58** [0.39–0.87]	0.65** [0.49–0.87]	0.60** [0.43–0.85]
Middle	0.65** [0.51–0.82]	0.84 [0.63–1.13]	0.42** [0.30–0.57]	0.45** [0.29–0.68]	0.57** [0.42–0.77]	0.51** [0.36–0.73]
Second richest	0.42** [0.32–0.54]	0.59** [0.42–0.83]	0.35** [0.25–0.48]	0.30** [0.17–0.53]	0.49** [0.37–0.66]	0.56** [0.37–0.85]
Richest	0.28** [0.22–0.35]	0.58* [0.38–0.88]	0.27** [0.19–0.39]	0.26** [0.12–0.54]	0.20** [0.14–0.29]	0.28** [0.16–0.49]
Place of residence						
Urban (R)						
Rural	1.86** [1.51–2.30]	1.06 [0.83–1.35]	1.97** [1.52–2.56]	1.35 [0.94–1.92]	1.42** [1.16–1.77]	1.05 [0.82–1.34]
Ecological belt						
Mountain (R)						
Hill	0.64** [0.51–0.81]	0.90 [0.70–1.15]	0.65** [0.49–0.85]	0.67* [0.49–0.91]	0.54** [0.37–0.79]	0.80 [0.54–1.18]
*Terai*	0.54** [0.43–0.69]	0.51** [0.37–0.72]	0.53** [0.40–0.71]	0.67 [0.43–1.03]	0.66* [0.46–0.94]	0.84 [0.53–1.33]
Household food security status						
Food secure (R)						
Mildly food insecure	NA	NA	1.40** [1.02–1.95]	0.86 [0.58–1.29]	1.36** [1.07–1.71]	0.98 [0.75–1.28]
Moderately food insecure	NA	NA	1.68** [1.28–2.21]	1.07 [0.75–1.53]	1.76** [1.37–2.26]	1.10 [0.82–1.47]
Severely food insecure	NA	NA	1.93** [1.39–2.67]	1.17 [0.77–1.77]	2.10** [1.43–3.12]	1.32 [0.86–2.01]
Access of drinking water						
Unimproved (R)						
Improved	0.90 [0.76–1.06]	1.19 [0.98–1.44]	0.67 [0.51–0.88]	0.96 [0.68–1.34]	0.72 [0.48–1.07]	0.82 [0.53–1.29]
Access of toilet						
Unimproved (R)						
Improved	0.50** [0.41–0.61]	0.83 [0.59–1.17]	0.65** [0.51–0.81]	1.27 [0.91–1.77]	0.49** [0.38–0.62]	0.64 ** [0.46–0.88]
*Maternal characteristics*						
Age of mother	1.05* [1.03–1.06]	0.97 [0.95–0.99]	1.03* [1.01–1.05]	0.97 [0.94–1.01]	1.02* [1.00–1.03]	0.99 [0.96–1.02]
Years of schooling of mother	0.87** [0.85–0.88]	0.92** [0.89–0.95]	0.92** [0.89–0.94]	1.00 [0.65–1.05]	0.91** [0.89–0.93]	0.98 [0.95–1.01]
Number of living children	1.65** [1.51–1.79]	1.12 [0.96–1.32]	1.39** [1.23–1.58]	1.05 [0.86–1.28]	1.50** [1.33–1.70]	1.17 [0.95–1.44]
Employment						
No (R)						
Yes	1.45** [1.21–1.74]	0.98 [0.80–1.21]	1.25 [0.96–1.62]	0.83 [0.59–1.17]	1.44** [1.20–1.73]	1.27* [1.01–1.60]
Mother BMI						
less than 18.5/underweight (R)						
18.5 and above	1.35** [1.16–1.58]	1.19 [0.98–1.44]	1.42** [1.10–1.83]	1.30 [0.93–1.81]	1.59** [1.23–2.04]	1.23 [0.94–1.60]
Mother anemia						
No (R)						
Yes	1.12 [0.97–1.30]	1.24* [1.04–1.48]	1.06 [0.86–1.30]	0.95 [0.74–1.23]	1.01 [0.84–1.24]	0.90 [0.72–1.14]
*Child characteristics*						
Age of child	2.11** [1.94–2.29]	2.24** [1.96–2.56]	1.80** [1.59–2.03]	2.58** [2.14–3.11]	1.61** [1.44–1.80]	1.58** [1.32–1.90]
Sex of child						
Boys (R)						
Girls	1.05 [0.91–1.21]	1.00 [0.85–1.17]	0.92 [0.77–1.10]	0.88 [0.71–1.10]	0.98 [0.81–1.20]	0.95 [0.76–1.17]
Birth order	1.17*** [1.13–1.21]	1.11* [1.02–1.20]	1.00 [1.00–1.01]	1.14 [1.03–1.26]	1.15** [1.09–1.21]	0.99 [0.89–1.10]
Size at the time of birth						
Average or larger (R)						
Below average	1.64** [1.37–1.946]	1.64** [1.34–2.00]	1.67** [1.29–2.18]	1.55** [1.19–2.02]	1.64** [1.31–2.05]	1.60** [1.25–2.04]
Anemia						
No (R)						
Yes	1.01 [0.87–1.17]	1.32** [1.12–1.56]	1.17 [0.95–1.43]	1.59** [1.25–2.02]	1.30** [1.06–1.61]	1.40** [1.12–1.75]

* *p* < 0.05; ** *p* < 0.01, see more on Additional files 4, 5 and 6

## Discussion

This paper outlines the prevalence of stunting and associated risk factors among children under five in Nepal. Study findings suggest that stunting in Nepal has decreased over the last decade (mean decline of − 1.92 in 2006 to − 1.29 in 2016). The Nepal government implemented the Multi-Sectoral Nutrition Plan (MSNP) in 2012 with the key objective of improving nutrition status among adolescent girls, pregnant and lactating women, and all children under 24 months. The MSNP is an evidence-based, cost-effective nutrition intervention that integrates both national and community priorities. The plan outlines the roles of key health, education, and agriculture sectors in implementing policies and strategies. Thus, decreases in stunting may be attributable to the implementation of the MSNP in Nepal [14, 15, 19, 20].

The study analyses identified significant determinants of stunting present across all three surveys; household wealth status, number of years of mother’s schooling, age of child, size of child at time of birth, and child anemia were all associated with stunting. Children from the poorest households were more likely to be stunted than children from middle income, richer, and richest households. This reflects findings from a systematic review conducted in sub-Saharan Africa by Akombi and colleagues [21, 22]. Likewise, a study based on the Bangladesh Demographic and Health Survey 2011 similarly found that wealth index was significantly associated with child stunting [21, 22]. The study findings also indicate that mothers with ten or more years of schooling were 37% less likely to have stunted children than mothers with no education. This reflects results from studies demonstrating less child stunting among mothers with more years of education [23–28]. Reduced child stunting among more educated mothers might reflect better feeding and care practices among these mothers [29].

Findings indicate that children aged 24–59 months were at higher risk for stunting than children aged 0–23. This is consistent with findings from similar studies [30–32], and may be explained by the protective effect of breastfeeding. While most children in Nepal are breastfed until 24 months, breastfeeding gradually declines with child age [33]. Children who had a low birth weight (less than 2.5 kg) were 1.5 times more likely to be stunted later in life than children of average or greater birth weight. The observed association between stunting and birth weight is consistent with other studies conducted in developing countries [34–38]. This link could be explained by the greater likelihood of low birth weight children to contract infections like diarrhea and acute respiratory infection. These infections may make the child more susceptible to later complications associated with stunting.

Child anemia was another determinant for stunting; results suggest that anemic children were 1.3 to 1.6 times more likely to be stunted than non-anemic children. Other studies have similarly found childhood anemia to be a significant determinant for stunting [39–42]. The link between anemia and stunting may be caused by anemia’s role in limiting children’s physical growth [43, 44] and iron supplementation could be used to reduce childhood stunting [45].

The findings of this study have policy implications as they provide a clear depiction of the trends and determinants of stunting from 3 nationally representative surveys over a 15 year period. Policy makers and planners can use evidence from this study to formulate feasible and culturally appropriate interventions to reduce stunting in Nepal. Though this paper explores determinants of stunting from the last 15 years of data, it is not free from limitations. The analyses are based on cross-sectional survey data; thus, we were unable to assess the causal relationship between stunting and socio-economic confounders.

## Conclusion

The prevalence of stunting in Nepal remains among the highest of developing countries. This study highlights risk factors for stunting, including household wealth quintile, number of years of mother’s schooling, child age, child size at birth, and child anemia. In addition to the policies and programs aimed at improving maternal and child nutrition, equal focus should be given to improving mothers’ education. Findings also suggest the importance of addressing income inequality when implementing nutrition interventions.

## Additional files

Additional file 1: **Table S1.** Prevalence of stunting (<−2SD) among children aged 0–59 months in 2006. (DOCX 16 kb)

Additional file 2: **Table S2.** Prevalence of stunting (<−2SD) among children aged 0–59 months in 2011. (DOCX 16 kb)

Additional file 3: **Table S3.** Prevalence of stunting (<−2SD) among children aged 0–59 months in 2016. (DOCX 16 kb)

Additional file 4: **Table S4.** Odd ratios of stunting (height for age < −2SD) in 2006. (DOCX 15 kb)

Additional file 5: **Table S5.** Odd ratios of stunting (height for age < −2SD) in 2011. (DOCX 14 kb)

Additional file 6: **Table S6.** Odd ratios of stunting (height for age < −2SD) in 2016. (DOCX 14 kb)

## Data Availability

The datasets used in this study are available from the corresponding author upon request.

## Abbreviations

BMI: Body Mass Index
HFIAS: Household Food Insecurity Scale
MSNP: Multi-Sectoral Nutrition Plan
NDHS: Nepal Demographic and Health Survey
OR: Odds Ratio
PPS: Probability proportion-to- size
SDG: Sustainable Development Goal
STATA: Software for Statistics and Data Science
