# Supplementary material for: Determinants of stunting among children aged 0–59 months in Nepal: findings from Nepal Demographic and health Survey, 2006, 2011, and 2016
Source: BMC Nutr. 2019 Aug 5;5:37. doi: 10.1186/s40795-019-0300-0 (PMC7050935; doi:10.1186/s40795-019-0300-0)
Supplement: Supplementary file 1 — Table S1. Prevalence of stunting (<−2SD) among children aged 0–59 months in 2006. (DOCX 16 kb) [file 40795_2019_300_MOESM1_ESM.docx]

Additional file 1:  *Prevalence of stunting (<-2SD) among children aged 0-59 months in 2006*

|  | Stunting (%) | Not stunting (%) | Total (%) | N |
| --- | --- | --- | --- | --- |
| Total | 49.3 | 50.7 | 100.0 | 5,083 |
| ***Household characteristics*** |  |  |  |  |
| **Family size** |  |  |  |  |
| Less than 5 | 43.2 | 56.8 | 100.0 | 1247 |
| 5 and above | 51.0 | 49.0 | 100.0 | 3836 |
| **Headship of the households** |  |  |  |  |
| Male | 48.7 | 51.3 | 100.0 | 4052 |
| Female | 50.9 | 49.1 | 100.0 | 1032 |
| **Caste/ethnicity** |  |  |  |  |
| Dalit | 56.5 | 43.5 | 100.0 | 788 |
| Muslim | 56.6 | 43.4 | 100.0 | 287 |
| *Janajati* | 44.9 | 55.1 | 100.0 | 1608 |
| Other *Terai* caste | 50.3 | 49.7 | 100.0 | 713 |
| Brahmin/chhetri | 47.8 | 52.2 | 100.0 | 1444 |
| Other | 48.5 | 51.5 | 100.0 | 244 |
| **Wealth quintile** |  |  |  |  |
| Poorest | 61.2 | 38.8 | 100.0 | 1286 |
| Second poorest | 54.4 | 45.6 | 100.0 | 1086 |
| Middle | 50.6 | 49.4 | 100.0 | 1032 |
| Second richest | 39.7 | 60.3 | 100.0 | 921 |
| Richest | 30.5 | 69.5 | 100.0 | 759 |
| **Place of residence** |  |  |  |  |
| Urban | 35.8 | 64.2 | 100.0 | 619 |
| Rural | 51.0 | 49.0 | 100.0 | 4465 |
| **Ecological Zone** |  |  |  |  |
| Mountain | 61.2 | 38.8 | 100.0 | 425 |
| Hill | 50.2 | 49.8 | 100.0 | 2100 |
| *Terai* | 46.2 | 53.8 | 100.0 | 2558 |
| **Household food security status** |  |  |  |  |
| Food secure | NA |  |  |  |
| Mildly food insecure | NA |  |  |  |
| Moderately food insecure | NA |  |  |  |
| Severely food insecure | NA |  |  |  |
| **Access of drinking water** |  |  |  |  |
| Unimproved | 51.2 | 48.8 | 100.0 | 1296 |
| Improved | 48.4 | 51.6 | 100.0 | 3787 |
| **Access of toilet** |  |  |  |  |
| Unimproved | 53.9 | 46.1 | 100.0 | 3658 |
| Improved | 36.9 | 63.1 | 100.0 | 1424 |
| ***Maternal characteristics*** |  |  |  |  |
| **Age of mother** |  |  |  |  |
| 15-19 | 33.6 | 66.4 | 100.0 | 333 |
| 20-24 | 45.4 | 54.6 | 100.0 | 1761 |
| 25-29 | 49.2 | 50.8 | 100.0 | 1595 |
| 30 and above | 58.6 | 41.4 | 100.0 | 1395 |
| **Years of schooling of mother** |  |  |  |  |
| No schooling | 57.3 | 42.7 | 100.0 | 3120 |
| 1-5 years schooling | 45.7 | 54.3 | 100.0 | 858 |
| 6-9 years schooling | 31.9 | 68.1 | 100.0 | 788 |
| 10 and above years of schooling | 20.1 | 79.9 | 100.0 | 318 |
| **Number of living children** |  |  |  |  |
| Up to 1 children | 34.0 | 66.0 | 100.0 | 1104 |
| 2 children | 46.0 | 54.0 | 100.0 | 1614 |
| 3 and more children | 58.3 | 41.7 | 100.0 | 2366 |
| **Mother Employment** |  |  |  |  |
| No | 42.7 | 57.3 | 100.0 | 1580 |
| Yes | 52.0 | 48.0 | 100.0 | 3503 |
| **Mother BMI** |  |  |  |  |
| less than 18.5/underweight | 47.2 | 52.8 | 100.0 | 3797 |
| 18.5 and above | 54.7 | 45.3 | 100.0 | 1284 |
| **Mother anemia** |  |  |  |  |
| No | 48.1 | 51.9 | 100.0 | 3027 |
| Yes | 50.9 | 49.1 | 100.0 | 2023 |
| ***Child characteristics*** |  |  |  |  |
| **Age of child** |  |  |  |  |
| Less than 6 months | 11.5 | 88.5 | 100.0 | 474 |
| 6-11 months | 23.65 | 76.35 | 100.0 | 484 |
| 12-23 months | 47.5 | 52.5 | 100.0 | 970 |
| 25-49 months | 59.2 | 40.8 | 100.0 | 3156 |
| **Sex of child** |  |  |  |  |
| Boys | 48.6 | 51.4 | 100.0 | 2604 |
| Girls | 49.7 | 50.3 | 100.0 | 2479 |
| **Birth order** |  |  |  |  |
| First | 40.6 | 59.4 | 100.0 | 1378 |
| Second | 44.9 | 55.1 | 100.0 | 1266 |
| Third and above | 56.1 | 43.9 | 100.0 | 2439 |
| **Size at the time of birth** |  |  |  |  |
| Average or larger | 46.9 | 53.1 | 100.0 | 4109 |
| Below average | 59.1 | 40.9 | 100.0 | 930 |
| **Anemia** |  |  |  |  |
| No | 53.6 | 46.4 | 100.0 | 2284 |
| Yes | 53.9 | 46.1 | 100.0 | 2191 |
