# Supplementary material for: Determinants of stunting among children aged 0–59 months in Nepal: findings from Nepal Demographic and health Survey, 2006, 2011, and 2016
Source: BMC Nutr. 2019 Aug 5;5:37. doi: 10.1186/s40795-019-0300-0 (PMC7050935; doi:10.1186/s40795-019-0300-0)
Supplement: Supplementary file 2 — Table S2. Prevalence of stunting (<−2SD) among children aged 0–59 months in 2011. (DOCX 16 kb) [file 40795_2019_300_MOESM2_ESM.docx]

Additional file 2:  *Prevalence of stunting (<-2SD) among children aged 0-59 months in 2011*

|  | Stunting (%) | Not stunting (%) | Total (%) | N |
| --- | --- | --- | --- | --- |
| Total | 40.5 | 59.5 | 100.0 | 2,485 |
| ***Household characteristics*** |  |  |  |  |
| **Family size** |  |  |  |  |
| Less than 5 | 38.9 | 61.1 | 100.0 | 693 |
| 5 and above | 41.1 | 58.9 | 100.0 | 1792 |
| **Headship of the households** |  |  |  |  |
| Male | 41.2 | 58.8 | 100.0 | 1828 |
| Female | 38.7 | 61.3 | 100.0 | 657 |
| **Caste/ethnicity** |  |  |  |  |
| Dalit | 47.0 | 53.0 | 100.0 | 440 |
| Muslim | 31.0 | 69.0 | 100.0 | 147 |
| *Janajati* | 40.4 | 59.6 | 100.0 | 794 |
| Other *Terai* caste | 45.9 | 54.1 | 100.0 | 230 |
| Brahmin/chhetri | 36.8 | 63.2 | 100.0 | 734 |
| Other | 41.0 | 59.0 | 100.0 | 140 |
| **Wealth quintile** |  |  |  |  |
| Poorest | 56.0 | 44.0 | 100.0 | 640 |
| Second poorest | 45.7 | 54.3 | 100.0 | 510 |
| Middle | 34.5 | 65.5 | 100.0 | 582 |
| Second richest | 30.5 | 69.5 | 100.0 | 421 |
| Richest | 25.8 | 74.2 | 100.0 | 332 |
| **Place of residence** |  |  |  |  |
| Urban | 26.7 | 73.3 | 100.0 | 217 |
| Rural | 41.8 | 58.2 | 100.0 | 2268 |
| **Ecological Zone** |  |  |  |  |
| Mountain | 52.9 | 47.1 | 100.0 | 196 |
| Hill | 42.1 | 57.9 | 100.0 | 991 |
| *Terai* | 37.4 | 62.6 | 100.0 | 1297 |
| **Household food security status** |  |  |  |  |
| Food secure | 33.2 | 66.8 | 100.0 | 1061 |
| Mildly food insecure | 41.2 | 58.8 | 100.0 | 305 |
| Moderately food insecure | 45.6 | 54.4 | 100.0 | 577 |
| Severely food insecure | 49.0 | 51.0 | 100.0 | 542 |
| **Access of drinking water** |  |  |  |  |
| Unimproved | 48.9 | 51.1 | 100.0 | 361 |
| Improved | 39.1 | 60.9 | 100.0 | 2123 |
| **Access of toilet** |  |  |  |  |
| Unimproved | 45.0 | 55.0 | 100.0 | 1415 |
| Improved | 34.6 | 65.4 | 100.0 | 1070 |
| ***Maternal characteristics*** |  |  |  |  |
| **Age of mother** |  |  |  |  |
| 15-19 | 27.9 | 72.1 | 100.0 | 170 |
| 20-24 | 39.8 | 60.2 | 100.0 | 908 |
| 25-29 | 38.7 | 61.3 | 100.0 | 740 |
| 30 and above | 46.7 | 53.3 | 100.0 | 667 |
| **Years of schooling of mother** |  |  |  |  |
| No schooling | 46.9 | 53.1 | 100.0 | 1256 |
| 1-5 years schooling | 41.7 | 58.3 | 100.0 | 442 |
| 6-9 years schooling | 32.1 | 67.9 | 100.0 | 464 |
| 10 and above years of schooling | 25.9 | 74.1 | 100.0 | 323 |
| **Number of living children** |  |  |  |  |
| Up to 1 children | 31.9 | 68.1 | 100.0 | 680 |
| 2 children | 38.5 | 61.5 | 100.0 | 751 |
| 3 and more children | 47.5 | 52.5 | 100.0 | 1054 |
| **Mother Employment** |  |  |  |  |
| No | 37.5 | 62.5 | 100.0 | 1116 |
| Yes | 42.9 | 57.1 | 100.0 | 1369 |
| **Mother BMI** |  |  |  |  |
| less than 18.5/underweight | 38.6 | 61.4 | 100.0 | 1935 |
| 18.5 and above | 47.1 | 52.9 | 100.0 | 469 |
| **Mother anemia** |  |  |  |  |
| No | 39.8 | 60.2 | 100.0 | 1476 |
| Yes | 41.2 | 58.8 | 100.0 | 902 |
| ***Child characteristics*** |  |  |  |  |
| **Age of child** |  |  |  |  |
| Less than 6 months | 19.4 | 80.6 | 100.0 | 228 |
| 6-11 months | 15.9 | 84.1 | 100.0 | 247 |
| 12-23 months | 34.8 | 65.2 | 100.0 | 489 |
| 25-49 months | 49.5 | 50.5 | 100.0 | 1522 |
| **Sex of child** |  | 100 |  |  |
| Boys | 41.4 | 58.6 | 100.0 | 1273 |
| Girls | 39.5 | 60.5 | 100.0 | 1212 |
| **Birth order** |  |  |  |  |
| First | 34.2 | 65.8 | 100.0 | 781 |
| Second | 39.2 | 60.8 | 100.0 | 592 |
| Third and above | 45.6 | 54.4 | 100.0 | 1112 |
| **Size at the time of birth** |  | 100 |  |  |
| Average or larger | 38.4 | 61.6 | 100.0 | 2061 |
| Below average | 50.7 | 49.3 | 100.0 | 424 |
| **Anemia** |  |  |  |  |
| No | 41.4 | 58.6 | 100.0 | 1172 |
| Yes | 45.2 | 54.8 | 100.0 | 1008 |
