# Supplementary material for: Determinants of stunting among children aged 0–59 months in Nepal: findings from Nepal Demographic and health Survey, 2006, 2011, and 2016
Source: BMC Nutr. 2019 Aug 5;5:37. doi: 10.1186/s40795-019-0300-0 (PMC7050935; doi:10.1186/s40795-019-0300-0)
Supplement: Supplementary file 3 — Table S3. Prevalence of stunting (<−2SD) among children aged 0–59 months in 2016. (DOCX 16 kb) [file 40795_2019_300_MOESM3_ESM.docx]

Additional file 3:  *Prevalence of stunting (<-2SD) among children aged 0-59 months in 2016*

|  | Stunting (%) | Not stunting (%) | Total (%) | N |
| --- | --- | --- | --- | --- |
| Total | 35.8 | 64.2 | 100.0 | 2,421 |
| ***Household characteristics*** |  |  |  |  |
| **Family size** |  |  |  |  |
| Less than 5 | 29.3 | 70.7 | 100.0 | 727 |
| 5 and above | 38.7 | 61.3 | 100.0 | 1693 |
| **Headship of the households** |  |  |  |  |
| Male | 36.2 | 63.8 | 100.0 | 1651 |
| Female | 35.0 | 65.0 | 100.0 | 769 |
| **Caste/ethnicity** |  |  |  |  |
| Dalit | 38.7 | 61.3 | 100.0 | 342 |
| Muslim | 37.2 | 62.8 | 100.0 | 164 |
| *Janajati* | 32.2 | 67.8 | 100.0 | 661 |
| Other *Terai* caste | 42.3 | 57.7 | 100.0 | 479 |
| Brahmin/chhetri | 34.6 | 65.4 | 100.0 | 629 |
| Other | 28.3 | 71.7 | 100.0 | 144 |
| **Wealth quintile** |  |  |  |  |
| Poorest | 49.2 | 50.8 | 100.0 | 496 |
| Second poorest | 38.7 | 61.3 | 100.0 | 528 |
| Middle | 35.7 | 64.3 | 100.0 | 549 |
| Second richest | 32.4 | 67.6 | 100.0 | 526 |
| Richest | 16.5 | 83.5 | 100.0 | 322 |
| **Place of residence** |  |  |  |  |
| Urban | 32.0 | 68.0 | 100.0 | 1280 |
| Rural | 40.2 | 59.8 | 100.0 | 1141 |
| **Ecological Zone** |  |  |  |  |
| Mountain | 46.8 | 53.2 | 100.0 | 170 |
| Hill | 32.3 | 67.7 | 100.0 | 876 |
| *Terai* | 36.7 | 63.3 | 100.0 | 1374 |
| **Household food security status** |  |  |  |  |
| Food secure | 29.2 | 70.8 | 100.0 | 991 |
| Mildly food insecure | 35.9 | 64.1 | 100.0 | 557 |
| Moderately food insecure | 42.0 | 58.0 | 100.0 | 623 |
| Severely food insecure | 46.5 | 53.5 | 100.0 | 250 |
| **Access of drinking water** |  |  |  |  |
| Unimproved | 43.3 | 56.7 | 100.0 | 112 |
| Improved | 35.5 | 64.5 | 100.0 | 2308 |
| **Access of toilet** |  |  |  |  |
| Unimproved | 48.9 | 51.1 | 100.0 | 581 |
| Improved | 31.7 | 68.3 | 100.0 | 1840 |
| ***Maternal characteristics*** |  |  |  |  |
| **Age of mother** |  |  |  |  |
| 15-19 | 37.4 | 62.6 | 100.0 | 194 |
| 20-24 | 32.5 | 67.5 | 100.0 | 863 |
| 25-29 | 34.8 | 65.2 | 100.0 | 767 |
| 30 and above | 41.6 | 58.4 | 100.0 | 597 |
| **Years of schooling of mother** |  |  |  |  |
| No schooling | 45.3 | 54.7 | 100.0 | 910 |
| 1-5 years schooling | 36.4 | 63.6 | 100.0 | 449 |
| 6-9 years schooling | 31.7 | 68.3 | 100.0 | 563 |
| 10 and above years of schooling | 22.7 | 77.3 | 100.0 | 498 |
| **Number of living children** |  |  |  |  |
| Up to 1 children | 28.0 | 72.0 | 100.0 | 725 |
| 2 children | 32.0 | 68.0 | 100.0 | 841 |
| 3 and more children | 46.2 | 53.8 | 100.0 | 855 |
| **Mother Employment** |  |  |  |  |
| No | 31.7 | 68.3 | 100.0 | 1240 |
| Yes | 40.2 | 59.8 | 100.0 | 1181 |
| **Mother BMI** |  |  |  |  |
| less than 18.5/underweight | 33.6 | 66.4 | 100.0 | 1901 |
| 18.5 and above | 44.5 | 55.5 | 100.0 | 452 |
| **Mother anemia** |  |  |  |  |
| No | 35.4 | 64.6 | 100.0 | 1271 |
| Yes | 35.8 | 64.2 | 100.0 | 1074 |
| ***Child characteristics*** |  |  |  |  |
| **Age of child** |  |  |  |  |
| Less than 6 months | 13.5 | 86.5 | 100.0 | 218 |
| 6-11 months | 18.9 | 81.1 | 100.0 | 251 |
| 12-23 months | 37.4 | 62.6 | 100.0 | 512 |
| 25-49 months | 41.6 | 58.4 | 100.0 | 1440 |
| **Sex of child** |  |  |  |  |
| Boys | 36.0 | 64.0 | 100.0 | 1258 |
| Girls | 35.7 | 64.3 | 100.0 | 1163 |
| **Birth order** |  | 100 |  |  |
| First | 31.4 | 68.6 | 100.0 | 791 |
| Second | 30.1 | 69.9 | 100.0 | 657 |
| Third and above | 43.3 | 56.7 | 100.0 | 973 |
| **Size at the time of birth** |  |  |  |  |
| Average or larger | 34.0 | 66.0 | 100.0 | 2030 |
| Below average | 45.5 | 54.5 | 100.0 | 390 |
| **Anemia** |  |  |  |  |
| No | 34.7 | 65.3 | 100.0 | 1022 |
| Yes | 40.9 | 59.1 | 100.0 | 1137 |
