# Supplementary material for: Determinants of stunting among children aged 0–59 months in Nepal: findings from Nepal Demographic and health Survey, 2006, 2011, and 2016
Source: BMC Nutr. 2019 Aug 5;5:37. doi: 10.1186/s40795-019-0300-0 (PMC7050935; doi:10.1186/s40795-019-0300-0)
Supplement: Supplementary file 4 — Table S4. Odd ratios of stunting (height for age < −2SD) in 2006. (DOCX 15 kb) [file 40795_2019_300_MOESM4_ESM.docx]

Additional file 4: Odd ratios of stunting (height for age <-2SD) in 2006

| **Background characteristics** | Odd ratios of stunting (height for age <-2SD) in 2006 | |
| --- | --- | --- |
| ***Household characteristics*** | Unadjusted (OR, P/ CI) | Adjusted (OR, P/ CI) |
| Family size | 1.37** [ 1.16 -1.62] | 1.15 [0.95 – 1.38] |
| **Headship of the households** |  |  |
| Male (R) |  |  |
| Female | 1.09 [0.91 -1.31] | 1.01 [0.82 - 1.24] |
| **Caste/ethnicity** |  |  |
| Dalit(R) |  |  |
| Muslim | 1.00 [0.74 - 1.35] | 1.21 [0.82 - 1.80] |
| *Janajati* | 0.63 [0.48 - 0.83] | 0.73 [0.53 – 0.99] |
| Other *Terai* caste | 0.78 [0.59 -1.03] | 0.96 [0.69 - 1.33] |
| Brahmin/chhetri | 0.70 [0.57 - 0.89] | 0.94 [0.69 - 1.26] |
| Other | 0.72 [0.35 -1.50] | 0.80 [0.36 - 1.77] |
| **Wealth quintile** |  |  |
| Poorest (R) |  |  |
| Second poorest | 0.76** [0.63 - 0.92] | 0.87 [0.65 – 1.18] |
| Middle | 0.65** [0.51 - 0.82] | 0.84 [0.63 – 1.13] |
| Second richest | 0.42** [0.32 - 0.54] | 0.59** [0.42 - 0.83] |
| Richest | 0.28** [0.22 - 0.35] | 0.58* [0.38 - 0.88] |
| **Place of residence** |  |  |
| Urban (R) |  |  |
| Rural | 1.86** [1.51 - 2.30] | 1.06 [0.83 - 1.35] |
| Ecological belt |  |  |
| Mountain (R) |  |  |
| Hill | 0.64** [0.51 - 0.81] | 0.90 [0.70 - 1.15] |
| *Terai* | 0.54** [0.43 - 0.69] | 0.51** [0.37 - 0.72] |
| **Household food security status** |  |  |
| Food secure (R) |  |  |
| Mildly food insecure | NA | NA |
| Moderately food insecure | NA | NA |
| Severely food insecure | NA | NA |
| **Access of drinking water** |  |  |
| Unimproved (R) |  |  |
| Improved | 0.90 [0.76 - 1.06] | 1.19 [0.98 - 1.44] |
| **Access of toilet** |  |  |
| Unimproved (R) |  |  |
| Improved | 0.50** [0.41 - 0.61] | 0.83 [0.59 - 1.17] |
| ***Maternal characteristics*** |  |  |
| **Age of mother** | 1.05* [1.03 - 1.06] | 0.97 [0.95 – 0.99] |
| **Years of schooling of mother** | 0.87** [0.85 - 0.88] | 0.92** [0.89 – 0.95] |
| **Number of living children** | 1.65** [1.51 -1.79] | 1.12 [ 0.96 - 1.32] |
| **Employment** |  |  |
| No (R) |  |  |
| Yes | 1.45** [1.21 - 1.74] | 0.98 [0.80 - 1.21] |
| **Mother BMI** |  |  |
| less than 18.5/underweight (R) |  |  |
| 18.5 and above | 1.35** [1.16 - 1.58] | 1.19 [0.98 - 1.44] |
| **Mother anemia** |  |  |
| No (R) |  |  |
| Yes | 1.12 [0.97 - 1.30] | 1.24* [1.04 - 1.48] |
| ***Child characteristics*** |  |  |
| Age of child | 2.11** [1.94 - 2.29] | 2.24** [1.96 - 2.56] |
| **Sex of child** |  |  |
| Boys (R) |  |  |
| Girls | 1.05 [0.91 - 1.21] | 1.00 [0.85 - 1.17] |
| **Birth order** | 1.17*** [1.13 - 1.21] | 1.11* [1.02 - 1.20] |
| **Size at the time of birth** |  |  |
| Average or larger (R) |  |  |
| Below average | 1.64** [1.37 – 1.946] | 1.64** [1.34 - 2.00] |
| **Anemia** |  |  |
| No (R) |  |  |
| Yes | 1.01 [0.87- 1.17] | 1.32** [1.12 – 1.56] |

* *p*<0.05; ** *p*<0.01;
