# Supplementary material for: Determinants of stunting among children aged 0–59 months in Nepal: findings from Nepal Demographic and health Survey, 2006, 2011, and 2016
Source: BMC Nutr. 2019 Aug 5;5:37. doi: 10.1186/s40795-019-0300-0 (PMC7050935; doi:10.1186/s40795-019-0300-0)
Supplement: Supplementary file 5 — Table S5. Odd ratios of stunting (height for age < −2SD) in 2011. (DOCX 14 kb) [file 40795_2019_300_MOESM5_ESM.docx]

Additional file 5: Odd ratios of stunting (height for age <-2SD) in 2011

| **Background characteristics** | Odd ratios of stunting (height for age <-2SD) in 2011 | |
| --- | --- | --- |
| ***Household characteristics*** | Unadjusted (OR, P/CI) | Adjusted (OR, P/CI) |
| Family size | 1.01 [ 0.86 -1.40] | 0.87 [0.62 – 1.21] |
| **Headship of the households** |  |  |
| Male (R) |  |  |
| Female | 0.90 [0.71 -1.14] | 0.85 [0.64 - 1.14] |
| **Caste/ethnicity** |  |  |
| Dalit(R) |  |  |
| Muslim | 0.50 [0.30 -0.85] | 0.55 [0.27 - 1.14] |
| Janajati | 0.76 [0.56 -1.04] | 0.89 [0.61 - 1.29] |
| Other Terai caste | 0.95 [0.63 -1.43] | 1.56 [1.04 - 1.33] |
| Brahmin/chhetri | 0.65 [0.51 - 0.85] | 0.80 [0.58 - 1.09] |
| Other | 0.78 [0.48 -1.29] | 0.96 [0.39 - 2.37] |
| **Wealth quintile** |  |  |
| Poorest (R) |  |  |
| Second poorest | 0.66** [0.50 - 0.88] | 0.58** [0.39 - 0.87] |
| Middle | 0.42** [0.30 - 0.57] | 0.45** [0.29 - 0.68] |
| Second richest | 0.35** [0.25 - 0.48] | 0.30** [0.17 - 0.53] |
| Richest | 0.27** [0.19 - 0.39] | 0.26** [0.12 - 0.54] |
| **Place of residence** |  |  |
| Urban (R) |  |  |
| Rural | 1.97** [1.52 - 2.56] | 1.35 [0.94 - 1.92] |
| Ecological belt |  |  |
| Mountain (R) |  |  |
| Hill | 0.65** [0.49 - 0.85] | 0.67* [0.49 - 0.91] |
| Terai | 0.53** [0.40 - 0.71] | 0.67 [0.43 - 1.03] |
| **Household food security status** |  |  |
| Food secure (R) |  |  |
| Mild food insecure | 1.40** [1.02 - 1.95] | 0.86 [0.58 - 1.29] |
| Moderately food insecure | 1.68** [1.28 - 2.21] | 1.07 [0.75 - 1.53] |
| Severely food insecure | 1.93** [1.39 – 2.67] | 1.17 [0.77 – 1.77] |
| **Access of drinking water** |  |  |
| Improved (R) |  |  |
| Unimproved | 0.67 [0.51 - 0.88] | 0.96 [0.68 - 1.34] |
| **Access of toilet** |  |  |
| Improved (R) |  |  |
| Unimproved | 0.65** [0.51 - 0.81] | 1.27 [0.91 - 1.77] |
| ***Maternal characteristics*** |  |  |
| **Age of mother** | 1.03* [1.01 - 1.05] | 0.97 [0.94 - 1.01] |
| **Years of schooling of mother** | 0.92** [0.89 - 0.94] | 1.00 [0.65 - 1.05] |
| **Number of living children** | 1.39** [1.23 - 1.58] | 1.05 [ 0.86 - 1.28] |
| **Employment** |  |  |
| No (R) |  |  |
| Yes | 1.25 [0.96 - 1.62] | 0.83 [0.59 - 1.17] |
| **Mother BMI** |  |  |
| less than 18.5/underweight (R) |  |  |
| 18.5 and above | 1.42** [1.10 - 1.83] | 1.30 [0.93 - 1.81] |
| **Mother anemia** |  |  |
| No (R) |  |  |
| Yes | 1.06 [0.86 - 1.30] | 0.95 [0.74 - 1.23] |
| ***Child characteristics*** |  |  |
| Age of child | 1.80** [1.59 - 2.03] | 2.58** [2.14 - 3.11] |
| **Sex of child** |  |  |
| Boys (R) |  |  |
| Girls | 0.92 [0.77 - 1.10] | 0.88 [0.71 - 1.10] |
| **Birth order** | 1.00 [1.00 - 1.01] | 1.14 [1.03 - 1.26] |
| **Size at the time of birth** |  |  |
| Average or larger (R) |  |  |
| Below average | 1.67** [1.29 - 2.18] | 1.55** [1.19 - 2.02] |
| **Anemia** |  |  |
| No (R) |  |  |
| Yes | 1.17 [0.95- 1.43] | 1.59** [1.25 – 2.02] |

* *p*<0.05; ** *p*<0.01;
