# Supplementary material for: Determinants of stunting among children aged 0–59 months in Nepal: findings from Nepal Demographic and health Survey, 2006, 2011, and 2016
Source: BMC Nutr. 2019 Aug 5;5:37. doi: 10.1186/s40795-019-0300-0 (PMC7050935; doi:10.1186/s40795-019-0300-0)
Supplement: Supplementary file 6 — Table S6. Odd ratios of stunting (height for age < −2SD) in 2016. (DOCX 14 kb) [file 40795_2019_300_MOESM6_ESM.docx]

Additional file 6: Odd ratios of stunting (height for age <-2SD) in 2016

| **Background characteristics** | Odd ratios of stunting (height for age <-2SD) in 2016 | |
| --- | --- | --- |
| ***Household characteristics*** | Unadjusted (OR, P/CI) | Adjusted (OR, P/CI) |
| Family size | 1.52** [ 1.22 -1.90] | 1.39* [1.06 - 1.83] |
| **Headship of the households** |  |  |
| Male (R) |  |  |
| Female | 0.95 [0.77 -1.17] | 1.06 [0.84 - 1.35] |
| **Caste/ethnicity** |  |  |
| Dalit(R) |  |  |
| Muslim | 0.94 [0.60 -1.47] | 1.04 [0.61 - 1.78] |
| Janajati | 0.75 [0.53 -1.08] | 1.01 [0.69 - 1.47] |
| Other Terai caste | 1.16 [0.81 -1.66] | 1.18 [0.74 - 1.91] |
| Brahmin/chhetri | 0.84 [0.60 -1.17] | 1.08 [0.72 - 1.61] |
| Other | 0.63 [0.37 -1.05] | 0.60 [0.26 - 1.36] |
| **Wealth quintile** |  |  |
| Poorest (R) |  |  |
| Second poorest | 0.65** [0.49 - 0.87] | 0.60** [0.43 - 0.85] |
| Middle | 0.57** [0.42 - 0.77] | 0.51** [0.36 - 0.73] |
| Second richest | 0.49** [0.37 - 0.66] | 0.56** [0.37 - 0.85] |
| Richest | 0.20** [0.14 - 0.29] | 0.28** [0.16 - 0.49] |
| **Place of residence** |  |  |
| Urban (R) |  |  |
| Rural | 1.42** [1.16 - 1.77] | 1.05 [0.82 - 1.34] |
| Ecological belt |  |  |
| Mountain (R) |  |  |
| Hill | 0.54** [0.37 – 0.79] | 0.80 [0.54 - 1.18] |
| Terai | 0.66* [0.46 – 0.94] | 0.84 [0.53 - 1.33] |
| **Household food security status** |  |  |
| Food secure (R) |  |  |
| Mild food insecure | 1.36** [1.07 - 1.71] | 0.98 [0.75 - 1.28] |
| Moderately food insecure | 1.76** [1.37 - 2.26] | 1.10 [0.82 - 1.47] |
| Severely food insecure | 2.10** [1.43 - 3.12] | 1.32 [0.86 - 2.01] |
| **Access of drinking water** |  |  |
| Improved (R) |  |  |
| Unimproved | 0.72 [0.48 - 1.07] | 0.82 [0.53 - 1.29] |
| **Access of toilet** |  |  |
| Improved (R) |  |  |
| Unimproved | 0.49** [0.38 – 0.62] | 0.64 ** [0.46 - 0.88] |
| ***Maternal characteristics*** |  |  |
| **Age of mother** | 1.02* [1.00 - 1.03] | 0.99 [0.96 - 1.02] |
| **Years of schooling of mother** | 0.91** [0.89 -0.93] | 0.98 [0.95 - 1.01] |
| **Number of living children** | 1.50** [1.33 -1.70] | 1.17 [ 0.95 - 1.44] |
| **Employment** |  |  |
| No (R) |  |  |
| Yes | 1.44** [1.20 - 1.73] | 1.27* [1.01 - 1.60] |
| **Mother BMI** |  |  |
| less than 18.5/underweight (R) |  |  |
| 18.5 and above | 1.59** [1.23 - 2.04] | 1.23 [0.94 - 1.60] |
| **Mother anemia** |  |  |
| No (R) |  |  |
| Yes | 1.01 [0.84 - 1.24] | 0.90 [0.72 - 1.14] |
| ***Child characteristics*** |  |  |
| Age of child | 1.61** [1.44 -1.80] | 1.58** [1.32 - 1.90] |
| **Sex of child** |  |  |
| Boys (R) |  |  |
| Girls | 0.98 [0.81 - 1.20] | 0.95 [0.76 - 1.17] |
| **Birth order** | 1.15** [1.09 - 1.21] | 0.99 [0.89 - 1.10] |
| **Size at the time of birth** |  |  |
| Average or larger (R) |  |  |
| Below average | 1.64** [1.31 - 2.05] | 1.60** [1.25 - 2.04] |
| **Anemia** |  |  |
| No (R) |  |  |
| Yes | 1.30** [1.06 – 1.61] | 1.40** [1.12 - 1.75] |

* *p*<0.05; ** *p*<0.01;
